# More Is Not Always Better: Local Models Provide Accurate Predictions of Spectral Properties of Porphyrins

**DOI:** 10.3390/ijms23031201

**Published:** 2022-01-21

**Authors:** Aleksey I. Rusanov, Olga A. Dmitrieva, Nugzar Zh. Mamardashvili, Igor V. Tetko

**Affiliations:** 1 G.A. Krestov Institute of Solution Chemistry of the Russian Academy of Sciences, 153045 Ivanovo, Russia; rusanov.a.i@mail.ru (A.I.R.); dmitrievao.a@yandex.ru (O.A.D.); ngm@isc-ras.ru (N.Z.M.); 2 Helmholtz Munich, Institute of Structural Biology, Deutsches Forschungszentrum für Gesundheit und Umwelt (GmbH), D-85764 Neuherberg, Germany; 3 BIGCHEM GmbH, D-85716 Unterschleißheim, Germany

**Keywords:** QSPR, Random Forest, local model, chromophores, porphyrins, absorbance maximum wavelength, molar extinction coefficient

## Abstract

The development of new functional materials based on porphyrins requires fast and accurate prediction of their spectral properties. The available models in the literature for absorption wavelength and extinction coefficient of the Soret band have low accuracy for this class of compounds. We collected spectral data for porphyrins to extend the literature set and compared the performance of global and local models for their modelling using different machine learning methods. Interestingly, extension of the public database contributed models with lower accuracies compared to the models, which we built using porphyrins only. The later model calculated acceptable RMSE = 2.61 for prediction of the absorption band of 335 porphyrins synthesized in our laboratory, but had a low accuracy (RMSE = 0.52) for extinction coefficient. A development of models using only compounds from our laboratory significantly decreased errors for these compounds (RMSE = 0.5 and 0.042 for absorption band and extinction coefficient, respectively), but limited their applicability only to these homologous series. When developing models, one should clearly keep in mind their potential use and select a strategy that could contribute the most accurate predictions for the target application. The models and data are publicly available.

## 1. Introduction

Porphyrins represent a unique class of heterocyclic tetrapyrrolic organic molecules which are classified as strong dyes (chromophore) due to their pronounced light-absorbing properties. Their unique optical properties were intensively studied in recent decades and found to have a wide range of applications in medicine [1] biological imaging [2,3], photocatalytic [4], analytical [5], industrial [6], nonlinear optics (NLO) [7], and molecular photovoltaics [8,9]. The presence of a highly conjugated system allows porphyrin to have intense absorption of light in the visible region with very unique UV-vis spectra. The main feature of porphyrin spectra is the presence of a very intense band at the 400 nm region (the so-called Soret band). It is known that the modification of the porphyrin macrocycle, namely, its meso-substitution, has a greater effect on the position and intensity of this band [10]. Consequently, the Soret band is a convenient and sensitive tool reflecting changes both in the structure of molecules and the effect of solvents on it. 

The development of new chromophores frequently critically depends on the expertise of the chemist and requires a large amount of time and synthetic efforts to synthesize new compounds with the desired optical and photophysical properties. Computational methods for predicting the optical properties of new porphyrins could allow them to be estimated in advance and reduce costs of synthesis. Such methods are actively developed in the field now, in particular based on quantum chemistry calculations, but frequently they have some significant limitations. A four-orbital model introduced by Gouterman successfully explains the presence of peaks in the absorption spectra of porphyrin and metal-free porphyrins [11]. However, this theory is unable to explain why the maximum positions in the absorption spectra remain almost unchanged when measured in different solvents for certain kinds of porphyrins. The semi-empirical quantum-chemical methods PPP-MO [12] and ZINDO/S [13] require calibration using an experimental dataset to achieve accurate wavelength predictions [14]. Time-dependent density functional theory (TD-DFT) [15,16,17] and ab initio calculations [18] require high level calculations to account for both dynamical and non-dynamical electron correlation, which are computational demanding and limit the practicality of such methods to single/few molecules [19]. The results of quantum-chemical calculation frequently deviate from the experimental data by 0.2–0.3 eV [20,21].

In recent years, quantitative structure–property relationship (QSPR) modeling has become a powerful tool for predicting the optical properties of chromophores [22,23,24,25,26,27,28]. The QSPR approach is based on the assumption that the macroscopic properties of chemical compounds depends on the calculated molecular characteristics of the compounds, which are called molecular descriptors. The advantage of this approach lies in the fact that once model is developed, it requires only the knowledge of the chemical structure and does not dependent on any experimental properties [29]. Accurate computational prediction of spectral properties of new porphyrins could allow us to design new molecules with desired properties using traditional combinatorial chemistry approaches or structures generated by deep neural networks [30]. However, since QSPRs are statistical approaches, the accuracy of developed models critically depends on the quality of data and of adequacy of the training set to the compound to be predicted. Moreover, one can use either local (by using structurally related compounds) or global models (developed with diverse sets of compounds). The advantages of each approach for the prediction of spectral properties of compounds need to be better carefully evaluated and have not been performed so far.

In this study, we tested a previous model of Joung et al. [27] as well as several new models developed with a large set of dyes and porphyrins collected from the literature to predict spectral properties of new compounds synthesized in our laboratory.

## 2. Material and Methods

The absorption spectra for compounds synthesised in our laboratory were obtained by spectrophotometer Cary-100 (Aglient, Santa Clara, CA, USA) in the dichloromethane (chemically pure).

### 2.1. Datasets

**Figure 1:**
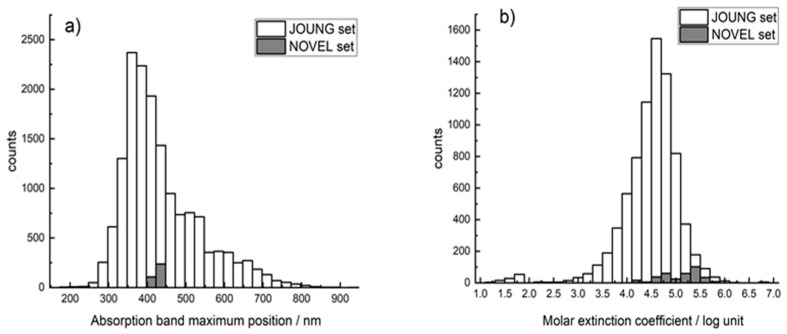
Histogram of the distribution of JOUNG and a NOVEL set of 335 porphyrins synthesized in our laboratory by absorption wavelengths (**a**) and the value of the extinction coefficient (**b**).

The initial analysis was performed using data from an article of Joung et al. [27] which contained optical properties of organic compounds collected from the literature which were described in [31] and were publicly available at FigShare link [32]. While Joung et al. [27] reported in their article 26,098 and 12,159 training set values for absorption band maximum position and extinction coefficient, respectively, the publicly accessible data at FigShare [32] contained only 17,294 and 8041 values for these optical properties, respectively. We excluded from these data organic compounds in the solid state, since our goal was to predict porphyrins in a liquid medium. Compounds which could not be processed by the On-line CHEmical database and Modeling environment (OCHEM) platform (very large and/or molecules with many rings for which calculation of descriptors failed) were also excluded. The remaining set (hereinafter JOUNG set) contained 6271 unique organic chromophores in 27 solvents, yielding 15,380 chromophore/solvent combinations for absorption band maximum position (Figure 1a) and 3753 unique organic chromophores in 25 solvents (7654 chromophore/solvent combinations) for molar extinction coefficient (Figure 1b). The database included various chromophore classes, but contained only 30 porphyrins.

**Figure 2:**
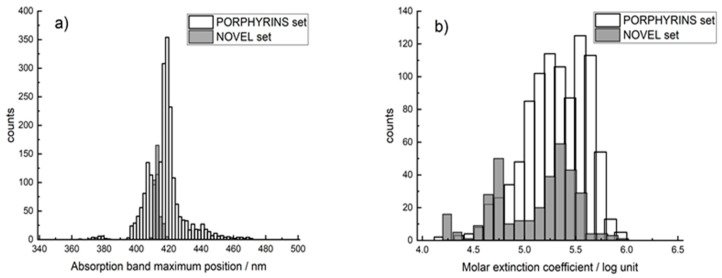
Histogram of the distribution of PORPHYRINS and a NOVEL set of 335 porphyrins synthesized in our laboratory by absorption wavelengths (**a**) and the value of the extinction coefficient (**b**).

The second set (hereinafter PORPHYRINS) was collected in this work from more than 30 publications. It included data for the first absorption peak of Soret porphyrins and their analogs (2241 unique compounds in dichloromethane), as well as their values of the logarithm of the molar extinction coefficient (946 unique compounds in dichloromethane). The database included the following macroheterocycles: chlorins, protoporphyrins, porphyrins, inverted porphyrins, their metal complexes and substituted at α- and β-positions by alkyl and aryl radicals, including halogens and radicals containing heteroatoms (Supplementary Data, Table S1). The Soret absorption wavelength values were in the region of 340–500 nm, with the majority of values in the range of 410–430 nm (Figure 2a). The values of the extinction coefficient were in range from 4.15 to 5.99, with most of them being in the range from 5 to 5.8 (Figure 2b).

The third analyzed set was a combination of JOUNG and PORPHYRINS (COMBINED).

The accuracy of models was tested using cross-validation results as well as on a set of 335 newly synthesized 2,8,12,18-tetramethyl-3,7,13,17-tetraalkyl-5,15-diphenylporph-yrins and 3,7,13,17-tetramethyl-2,8,12,18-tetraalkyl-5,15-diphenylporphyrins, as well as their zinc complexes, which were not present in any of the previous sets and were also not previously published by us (NOVEL set). The procedures for the synthesis of these compounds are described in the Experimental protocol section of the Supplementary Data while structural and optical properties are available in the Supplementary Excel file.

### 2.2. Methods

Quantitative models were developed using a variety of combinations of learning methods with a different set of descriptors, which were available in On-line Chemical Database and Modeling Environment (OCHEM) [33]. The default parameters of these algorithms as specified in OCHEM were used. Amid a preliminary analysis, we found that Random Forest Regression (RFR) [34] consistently contributed better results and therefore RFR was used for all analyses reported in this study. All descriptor packages available in OCHEM were used to provide a variety of chemical structure representations for spectral properties modeling. Amid them, several packages, namely ISIDA fragmentor descriptors [35], MOLD2 descriptors [36], alvaDesc [37], and SIRMS descriptors [38] consistently contributed models with the highest performances. Most of these packages used 2D representation of chemical compounds while the Corina program [39] was used to perform 2D to 3D conversion for the alvaDesc [37]. In addition to models based on descriptors, we also used Transformer Convolutional Neural Network [40], which is a representation learning method operating directly with text representation (SMILES [41]) of chemical structures. All descriptor packages and modelling methods were used with default values of parameters as described in details on the OCHEM website [42].

Five-fold cross-validation [43] was used to develop models. Once models for individual descriptor packages were developed, we selected those with the highest performance for the training set and used them to build a consensus, which was an average of individual models following methodology developed in our earlier studies [44,45,46]. The statistical parameters calculated by the consensus model were used to estimate predictive performance of machine learning methods.

### 2.3. Statistical Parameters

The quality of models was estimated using the squared correlation coefficient (*R*^2^) Equation (1) and root mean square error (RMSE) Equation (2):

(1) $$R^{2}=\frac{\sum_{i=1}^{n}\left(y_{pred,i}-{\underline{y}}_{pred}\right)\times \left(y_{exp,i}-{\underline{y}}_{exp}\right)}{\sum_{i=1}^{n}{\left(y_{pred,i}-{\underline{y}}_{pred}\right)}^{2}\times \sum_{i=1}^{n}{\left(y_{exp,i}-{\underline{y}}_{exp}\right)}^{2}}$$

(2) $$RMSE=\sqrt{\frac{1}{n}\overset{n}{\underset{i=1}{\sum }}{\left(y_{pred,i}-y_{exp,i}\right)}^{2}}$$

where *n* is the number of data points; *y_exp,i_* is the experimental and *y_pred,i_* is the predicted value of the analyzed data point *i*.

## 3. Results and Discussion

### Model Development and Testing

Our initial attempt was to predict the optical properties of the porphyrins from the NOVEL set using the model published by Joung et al. [27]. This model was accessed on the website (http://deep4chem.korea.ac.kr, accessed date is 30 December 2021). The results of predicting the positions of the Soret band maximum and the values of the extinction coefficient demonstrated low correlation between the predicted and experimental values (Figures S1 and S2) and were RMSE = 200 (*R*^2^ = 0.01) and RMSE = 0.89 (*R*^2^ = 0.1) for the maximum absorption and extinction coefficient of porphyrins, respectively (see Table 1 and Table 2). Thus, the published model could not predict the optical properties of porphyrins. 

**Figure 3:**
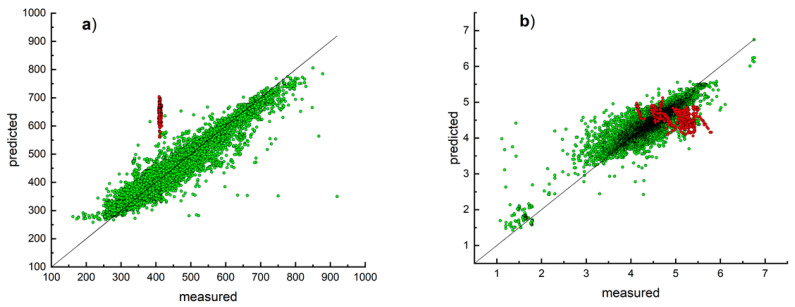
Distribution of the experimental and predicted values of the position of the absorption band (**a**) and values of the extinction coefficient (**b**) using models based on the JOUNG set. The green and red colors correspond to the training set data and test set data of 335 compounds, respectively.

As it was mentioned in the Data section, the JOUNG set contained only part of data published in Joung et al. [27]. To verify whether we can reproduce results of the original model of Joung et al. [27] with OCHEM tools, we developed QSPR models based on the JOUNG using the RFR method and different sets of descriptors. A 5-fold cross-validation was used to estimate accuracy of developed models. The initial calculations were performed with and without parameterization of the solvent using procedure described elsewhere [47]. The models with the best statistical parameters were chosen to create the consensus models as average of these individual models. We observed the same effect as in the previous study [47], namely that solvent parameterization did not provide significantly better results. For example, the mean difference between RMSE of consensus models for prediction with the parameterization of solvent and without it was 0.6 nm for the JOUNG set which was within the standard mean error of the model (Table S2). Since the difference was within the error range of the model accuracies, we decided to skip the use of solvent parameterization in the further analysis for absorption coefficient. The consensus model calculated correlation coefficient *R*^2^ = 0.90 and RMSE = 31.5 nm, which was similar to that (*R*^2^ = 0.926, RMSE = 31.6 nm) obtained by the authors for the test set compounds (10% of data). It should be mentioned that results of the 5-fold cross-validation protocol used in our study (20% of data were removed from the model and predicted based on the model training with remaining 80% of compounds; procedure was repeated 5 times and results for 20% excluded compounds were averaged) were more strict than the test set protocol reported by Joung et al. (90% of compounds were used for model hyperparameter tuning, training and validation; the performance was reported for 10% of left compounds). Similar to the original model developed by the authors, the consensus model also showed a low accuracy (*R*^2^ = 0.12 and RMSE = 204) for the NOVEL set (see also Table S2 and Figure 3a). Thus, the prediction of the absorption band based on the original model developed by Joung et al. or data from their study had a low accuracy for porphyrins.

Similar results were calculated for prediction of the extinction coefficient of chromophores and the consensus model developed using the JOUNG set provided low accuracy (*R*^2^ = 0.62, RMSE = 0.84) for prediction of the NOVEL set compounds, which was similar to that obtained with their original model (See Table 2 and Figure 3b). Similarly for absorption coefficient, an includance of the parametrization of solvent did not improve models and was not used in further studies.

The reason for the failure of models built on the JOUNG data could be due to the low number of porphyrins in these sets (only 30 out of 15,380), which thus did not cover the chemical space of porphyrins.

To improve the prediction results, we extended the JOUNG dataset with the PORPHYRINS set to form the COMBINED set (Table 1, Table 2 and Table S3). Like in the study with JOUNG dataset, the models with highest accuracy for this set were used to develop the consensus models. Consensus models improved the accuracy of predicting the position of the absorption band to RMSE = 21 nm and the extinction coefficient RMSE = 0.54 for the NOVEL set. The extension of the JOUNG dataset to include porphyrins provided a global model, which was covering various classes of molecules. A combination of the JOUNG with PORPHYRINS increased the accuracy of the resulting consensus model for the JOUNG subset (we calculated statistical parameters for compounds from this subset of the COMBINED set). The accuracy of the model for the PORPHYRINS subset was higher (RMSE = 10.3 vs. RMSE = 31.9) than that for the JOUNG set (Table 1 and Table 2). The same tendency was observed for the extinction coefficient, but differences in statistical parameters were smaller. This result indicated that likely the quality of experimental data for the PORPHYRINS set was higher than that for the JOUNG set. By mixing low and highly accurate data, we could improve less accurate data, but at the same time, could decrease the quality of the model for more accurate ones. Therefore, we decided to develop local models using the PORPHYRINS set only.

The same methodology was used to develop models using only the PORPHYRINS. The models for both absorption band and extinction coefficients calculated higher 5CV statistical parameters than those calculated for respective subsets when they were used as part of the COMBINED set (Table S4). The developed consensus models improved the prediction of the position of the Soret band and extinction coefficients of the NOVEL set as test set compounds to RMSE = 2.61 nm (Table 1) and RMSE = 0.52 (Table 2), respectively. Thus, the development of a local model just for porphyrins as compared to the development of a global model for various dyes provided higher cross-validation accuracy for this chemical class of compounds as well as better accuracy for prediction of the NOVEL set. Although we observed an improvement of the model for prediction of extinction coefficient, the accuracy of its prediction was not satisfactory and the model with RMSE of 0.5 could hardly have any practical value.

The prediction error for the NOVEL set of 335 compounds RMSE = 2.61 nm was lower than the 5CV RMSE = 5.4 nm estimated for the PORPHYRINS set. This was a very nice result, but the experimental accuracy of the absorption band was estimated in our laboratory to be about 0.5 nm. Thus, the predicted error was about five times larger than the experimental one. For the prediction of extinction coefficient, which was typically measured with accuracy of 0.01, the discrepancy between prediction and experimental errors was about 20 times. Considering that all data for the NOVEL set were all measured in our laboratory, we were interested in determining whether we could get a better model for them.

**Figure 4:**
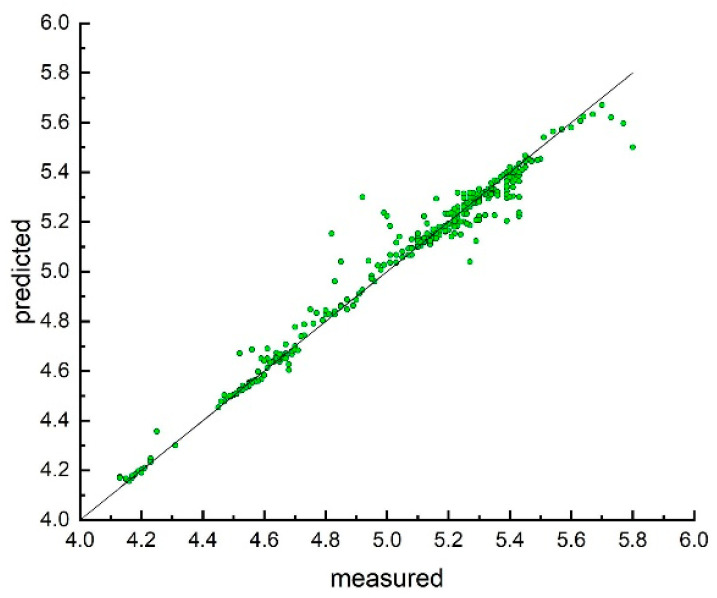
Distribution of the experimental and predicted values of the extinction coefficient calculated by consensus model developed with *n* = 335 compounds experimentally measured in this work.

**Figure 5:**
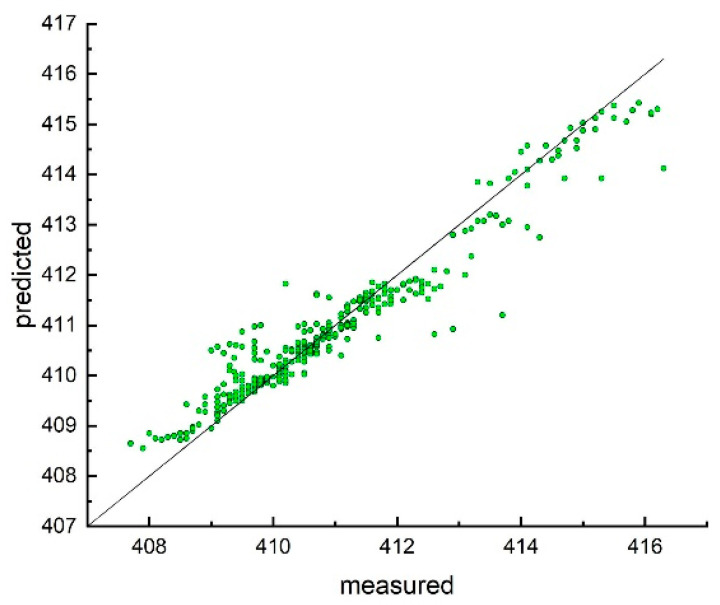
Distribution of the experimental and predicted values of the absorption maximum position calculated by consensus model developed with *n* = 335 compounds experimentally measured in this work.

Therefore, we used the same methodology as in the previous studies and calculated excellent consensus models for both properties for the NOVEL set (*n* = 335) estimated using the 5CV protocol (Table 1 and Table 2 and Figure 4 and Figure 5).

A possible reason for such good accuracy of both these models could be the minimum noise in the data, since all measurements were performed within the same laboratory using the same equipment. On the other hand, the compounds were homologous series and just differed in functional groups in the positions of the phenyl rings, as well as in the long alkyl chains in the beta-positions.

Thus, the development of models based on the homologous series of compounds provided the best accuracy for these data. At the same time, of course, models developed with such restricted chemical series can not be used to predict compounds from other dyes, which are structurally different. The model for absorption maximum position had a range of experimental values in the 408–418 nm region (see also Figure 1 and Figure 2) and could not extrapolate to values outside of this region. It calculated RMSE = 101.2 ± 0.8 and 14.4 ± 0.4 for prediction of dyes from JOUNG and PORPHYRINS sets, respectively. A smaller RMSE for the PORPHYRINS sets reflected a higher structural similarity of NOVEL set compounds as well as narrower range of absorption maximum position values for PORPHYRINS. Similarly, the model for extinction coefficient, which was based on the data coming from our laboratory, failed to predict these both sets too and calculated RMSE of 0.92 and 0.41 for JOUNG and PORPHYRINS, respectively. It should be mentioned that the majority of the predictions for both models were identified as out of the applicability domain [43], and thus the models correctly flagged such predictions as inconsistent with the training set data. Thus, the developed local models based on homologous series could be only applicable to these series. Contrary to that, the models developed using the PORPHYRINS sets are expected to predict a much wider class of porphyrins.

**Figure 6:**
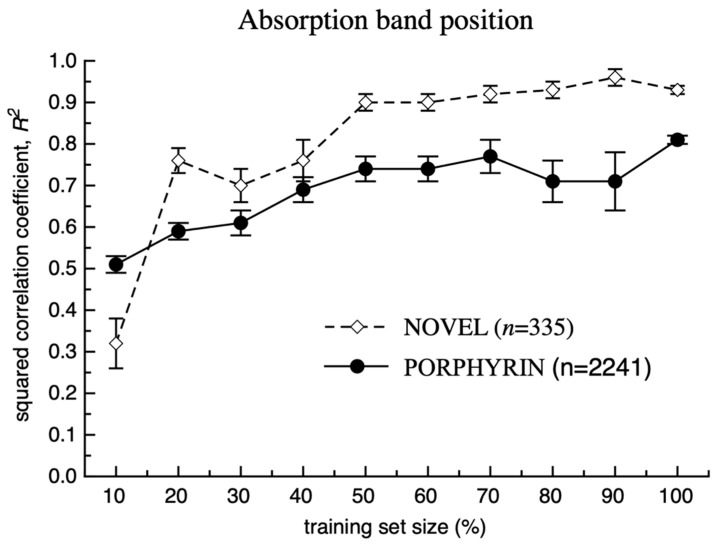
Statistical coefficients calculated for the prediction of the test set compounds that were not part of the respective training sets for modelling of the absorption band maximum position (see also Supplementary Data, Tables S5 and S6). 5CV values were reported for 100% training set size.

**Figure 7:**
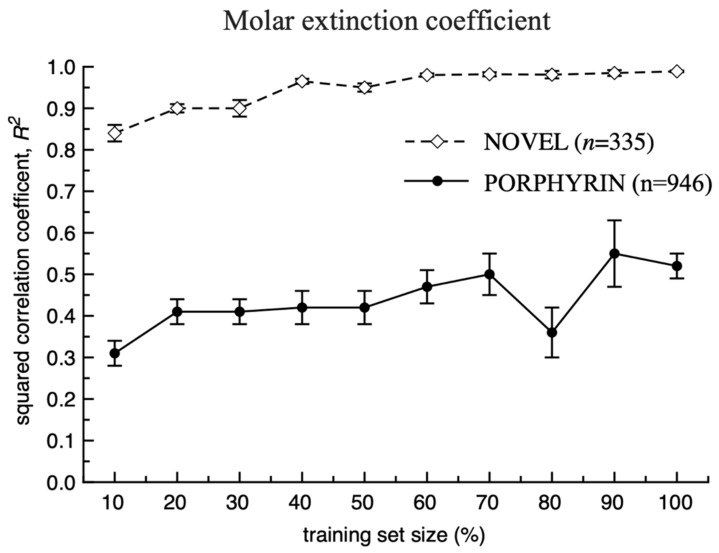
Statistical coefficients calculated for the prediction of the test set compounds that were not part of the respective training sets for modelling of the molar extinction coefficient (see also Supplementary Data, Tables S5 and S6). 5CV values were reported for 100% training set size.

In the last study, we investigated the influence of the size of the training set for the accuracy of the model for porphyrins. Subsets of compounds were randomly sampled from respective PORPHYRIN and NOVEL sets and were used to predict the remaining compounds from the same sets that were not used for model development (see Figure 6 and Figure 7 as well as supplementary Tables S5 and S6). With the increase of the training set size, the smaller numbers of compounds were left for testing which resulted in higher calculated errors bars. The performance of models for 100% data used as a training set was estimated using 5CV. 

For both spectral properties, an increase of the training dataset sizes steadily increased the squared correlation coefficient, *R*^2^ for the test sets. The accuracy of the models for the prediction of more diverse PORPHYRIN sets were lower compared to those calculated for the NOVEL set using the same percentage of the training set data. The squared correlation coefficients for the NOVEL set using 30–40% of data were similar to those calculated using 70–100% training set data of the PORPHYRIN set. The higher values for the NOVEL set could be explained by smaller structural diversity of compounds and thus higher density of data points allowing to adequately estimate the influence of various substituents on the variation of this coefficient. Likely by further increasing the size of the PORPHYRIN set with additional data, we could reach the same values of the squared correlation coefficient obtained for the NOVEL set.

However, in the case of the extinction coefficient, there was a different behaviour and we could observe a big gap in the performances of models developed with PORPHYRIN and NOVEL sets. Thus, a further increase in the amount of literature data for this coefficient is unlikely to result in the same accuracy of the model as we calculated using the NOVEL set.

The reason for the low prediction accuracy of the molecular extinction coefficient based on the literature data could be inconsistencies and errors when collecting this parameter from various sources. These errors depend on the sensitivity of the measurement devices, e.g., type of the used spectrophotometer and the scales on which the compounds were weighed, but as well as on rounding and possibly even simple arithmetic errors when calculating the extinction coefficient from the experimental data. At the same time, if the same equipment as well as the same protocol were strictly used for its measurement within the same laboratory, one could expect much higher accuracy and consistency of data which could result in excellent models with high statistical parameters, as reported in this study.

Thus, in this work, we first analyzed the prediction accuracy of published models to predict spectral properties of porphyrins synthesized in our laboratory (NOVEL set, *n* = 335). We found a low performance of both published (http://deep4chem.korea.ac.kr, accessed date is 30 December 2021) as well as models re-developed by us using the publicly available data deposited by the authors (JOUNG set). The RMSE for the prediction of maximum absorption band were in range of 200 nm while for the extinction coefficient RMSE of 0.8–0.9 log units were observed. The low performance of these models was attributed to a small number of porphyrins (*n* = 30) in the training sets. 

An extension of published sets by including porphyrins (COMBINED set) improved results for both spectral properties and RMSE = 21 and 0.54 were calculated for these properties for the NOVEL set. A development of local models using only PORPHYRINS set (*n* = 2241 for absorption and *n* = 946 for extinction coefficient) provided significant improvement of the accuracy of models (RMSE = 2.61) to predict the absorption band, but the accuracy of models for the extinction coefficient practically did not change (RMSE = 0.52). 

Interestingly, a development of models using the 335 compounds from the NOVEL set contributed highly predictive models with significantly higher accuracy (RMSE = 0.5 for absorption and RMSE = 0.042 for extinction coefficient). Since models developed using NOVEL set were based on compounds with limited chemical diversity (2,8,12,18-tetramethyl-3,7,13,17-tetraalkyl-5,15-diphenylporphyrins 3,7,13,17-tetrame-thyl-2,8,12,18-tetraalkyl-5,15-diphenylporphyrins, as well as their zinc complexes), they failed to predict molecules from the PORPHYRINS and JOUNG set, since most of the predictions were outside of the applicability domain of this model.

## 4. Conclusions

In this study, we contributed QSPR models for predicting the optical properties of porphyrins as well as reported synthesis protocols and experimental values for *n* = 335 porphyrins which are publicly available at http://ochem.eu/article/140403 (accessed date is 30 December 2021). We showed that a better strategy for this chemical class was to develop local models for porphyrins rather than to extend diverse sets of dyes with additional spectral properties of these compounds. While we could successfully model the Soret band, we could not obtain models with good accuracy to predict the extinction coefficient when using literature data. The failure to model the second property could be attributed to the experimental inconsistency of data obtained from various sources. Indeed, we obtained excellent models for both studied properties when using experimental data (NOVEL set) measured in our laboratory. Unfortunately, because of the very limited chemical diversity, models based on the NOVEL set have a limited applicability domain. 

Thus, when analyzing spectral properties of chemical dyes, a possibility to develop local models to cover the studied class of molecules should not be overlooked. While such models may not cover the whole chemical space of dyes, they could be adequate to accurately predict the investigated compounds in particular for properties, such as extinction coefficient, which strongly depend on the used experimental protocol. An attempt to combine in one set inconsistent data could result in a low quality model. More is not always better!

The developed QSPR models for porphyrins can be used to predict their optical properties before they are actually synthesized. This could help to identify compounds with desired sets of properties, significantly reduce development costs, and to accelerate the development of new functional optical materials for electronic and optoelectronic applications.

## Figures and Tables

**Table 1:** Statistical parameters of models developed using different training sets for prediction of absorption maximum band.

Data Set	Training Set, 5CV			Prediction of NOVEL Set, *n* = 335	
	*n*	*R* ^2^	RMSE	*R* ^2^	RMSE
Published model of Joung et al. [27]	26,098	0.926 ^a^	31.6 ^a^	0.01	200
JOUNG	15,380	0.904 ± 0.003	31.5 ± 0.5	0.12 ± 0.02	204 ± 2
COMBINED	17,621	0.9 ± 0.003	30.1 ± 0.5	0.03 ± 0.01	21 ± 1
COMBINED: JOUNG subset ^a^	15,380	0.902 ± 0.003	31.9 ± 0.5		
COMBINED: PORPHYRINS subset ^ab^	2241	0.43 ± 0.05	10.3 ± 0.7		
PORPHYRINS	2241	0.8 ± 0.01	5.4 ± 0.2	0 ± 0.005	2.61 ± 0.1
NOVEL set	335	0.93 ± 0.01	0.5 ± 0.03		

^a^ The results reported by Joung et al. [27]. ^b^ Statistical results were calculated for a respective subset of compounds from the COMBINED set.

**Table 2:** Statistical parameters of models developed using different training sets for prediction of the extinction coefficient.

Data Set	Training Set, 5CV			Prediction of NOVEL Set, *n* = 335	
	*n*	*R* ^2^	RMSE	*R* ^2^	RMSE
Published model of Joung et al. [27]	12,159	0.795 ^a^	0.24 ^a^	0.10	0.89
JOUNG	7654	0.767 ± 0.009	0.286 ± 0.005	0.62 ± 0.02	0.84 ± 0.02
COMBINED	8600	0.806 ± 0.007	0.279 ± 0.005	0 ± 0.006	0.54 ± 0.02
COMBINED: JOUNG subset ^a^	7654	0.765 ± 0.01	0.286 ± 0.005		
COMBINED: PORPHYRINS subset ^ab^	946	0.49 ± 0.03	0.218 ± 0.006		
PORPHYRINS	946	0.52 ± 0.02	0.209 ± 0.006	0 ± 0.004	0.52 ± 0.02
NOVEL set	335	0.989 ± 0.002	0.042 ± 0.004		

^a^ The results reported by Joung et al. [27]. ^b^ Statistical results were calculated for a respective subset of compounds from the COMBINED set.

## Data Availability

The Supplementary Materials contain synthesis protocols and experimental data for 335 novel compounds synthesized in this work as well as data tables supporting statistical analysis provided in this study.

## Supplementary Materials

The following supporting information can be downloaded at: www.mdpi.com/article/10.3390/ijms23031201/s1.
