# Supplementary material for: More Is Not Always Better: Local Models Provide Accurate Predictions of Spectral Properties of Porphyrins
Source: Int J Mol Sci. 2022 Jan 21;23(3):1201. doi: 10.3390/ijms23031201 (PMC8835262; doi:10.3390/ijms23031201)
Supplement: Supplementary file 1 [file ijms-23-01201-s001.zip › Supplementary_Data.pdf]

Supplementary materials

Table S1: Examples of different macroheterocycles included in the PORPHYRINS set.

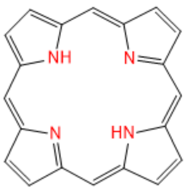	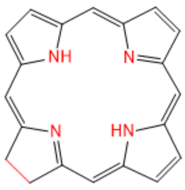	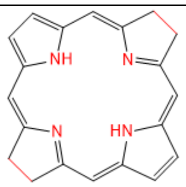
**Porphyrins and their complexes**<br>**2030**	**Chlorins**<br>**109**	**Bacteriochlorins**<br>**32**
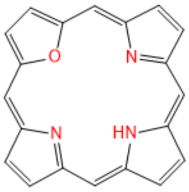	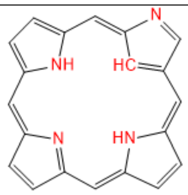	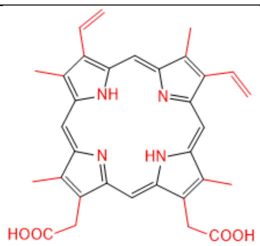
**Oxo-porphyrins**<br>**20**	**Inverted porphyrins**<br>**48**	**Protoporphyrins**<br>**21**
**Among them**		
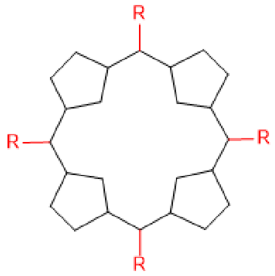	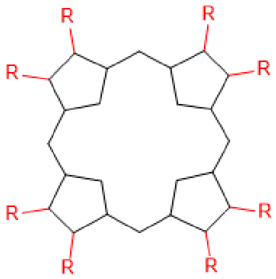	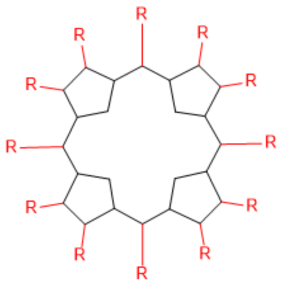
α-substituted	β-substituted	α,β-substituted
R = alkyl, aryl, halogen, groups and radicals with heteroatoms,etc		
**In phenyl-substituted porphyrins**		
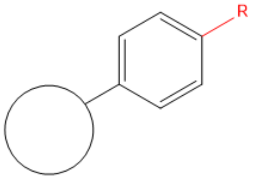	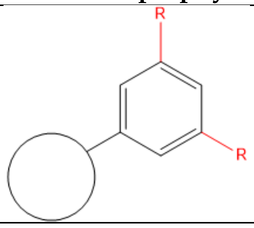	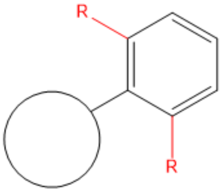
**4-phenyl**	**3/3,5-phenyl**	**2/2,6-phenyl**
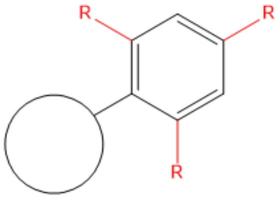	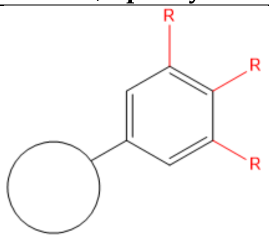	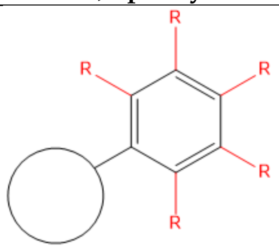
**2,4/2,4,6-phenyl**	**3,4/3,4,5-phenyl**	**pentsa-phenyl**
R = alkyl, aryl, halogen, groups and radicals with heteroatoms,etc		

Table S2: Statistical coefficients calculated for the QSPR model developed with JOUNG set

Absorption maxima position					Molar extinction coefficient				
**Descriptors**	Training, n=1530		Test, n=335		Descriptors	Training, n=7654		Test, n=335	
	R^2	RMSE/nm	R^2	RMSE/nm		R^2	RMSE/log unit	R^2	RMSE/log unit
Fragmentor	0.875 ± 0.004	35.5 ± 0.6	0.1 ± 0.02	205 ± 3	ALogPS	0.723 ± 0.01	0.311 ± 0.005	0.61 ± 0.04	0.65 ± 0.02
JPlogP	0.851 ± 0.004	39.1 ± 0.5	0.11 ± 0.02	208 ± 2	Fragmentor	0.73 ± 0.01	0.303 ± 0.005	0 ± 0.01	0.96 ± 0.02
MOLD2	0.837 ± 0.004	41 ± 0.5	0.1 ± 0.02	200 ± 2	JPlogP	0.7 ± 0.01	0.323 ± 0.006	0.16 ± 0.03	0.66 ± 0.02
QNPR	0.857 ± 0.004	38.2 ± 0.5	0.12 ± 0.02	203 ± 4	SIRMS	0.73 ± 0.01	0.306 ± 0.006	0.56 ± 0.03	1.1 ± 0.02
RDKIT	0.827 ± 0.005	42.4 ± 0.6	0.08 ± 0.03	190 ± 2	StructuralAlerts	0.69 ± 0.01	0.324 ± 0.006	0.68 ± 0.02	0.88 ± 0.02
SIRMS	0.875 ± 0.004	35.7 ± 0.5	0.13 ± 0.03	189 ± 3	QNPR	0.7 ± 0.01	0.321 ± 0.006	0.05 ± 0.02	0.7 ± 0.02
alvaDesc	0.854 ± 0.004	38.8 ± 0.5	0.14 ± 0.03	179 ± 2	RDKIT	0.7 ± 0.01	0.326 ± 0.005	0.35 ± 0.03	0.66 ± 0.02
Transformer CNN	0.902 ± 0.003	31.5 ± 0.5	0 ± 0.003	232 ± 1	Transformer CNN	0.74 ± 0.01	0.299 ± 0.007	0.66 ± 0.02	0.71 ± 0.02
Consensus model					Consensus model				
	0.904 ± 0.003	31.5 ± 0.5	0.12 ± 0.02	204 ± 3		0.767 ± 0.009	0.286 ± 0.005	0.62 ± 0.02	0.84 ± 0.02

Eight models providing the highest squared correlation coefficient, R2, for both analyzed properties are shown.

Table S3: Statistical coefficients calculated for the QSPR model for the COMBINED set.

Absorption maxima position					Molar extinction coefficient				
Descriptors	Training, n=17621		Test, n =335		Descriptors	Training, n=8600		Test, n=335	
	R^2	RMSE/nm	R^2	RMSE/nm		R^2	RMSE/log unit	R^2	RMSE/log unit
Fragmentor	0.874 ± 0.004	33.4 ± 0.5	0.013 ± 0.007	19 ± 1	ALogPS	0.771 ± 0.007	0.304 ± 0.005	0 ± 0.005	0.53 ± 0.02
JPlogP	0.846 ± 0.004	37.2 ± 0.5	0 ± 0.008	14.1 ± 0.7	Fragmentor	0.778 ± 0.008	0.297 ± 0.005	0 ± 0.003	0.56 ± 0.02
MOLD2	0.826 ± 0.005	39.7 ± 0.5	0.02 ± 0.009	31 ± 2	JPlogP	0.746 ± 0.008	0.318 ± 0.0055	0 ± 0.003	0.49 ± 0.02
QNPR	0.856 ± 0.004	35.9 ± 0.5	0.014 ± 0.007	21 ± 1	PyDescriptor	0.752 ± 0.008	0.317 ± 0.005	0.02 ± 0.02	0.5 ± 0.02
RDKIT	0.823 ± 0.004	40.1 ± 0.5	0.019 ± 0.01	27 ± 1	RDKIT	0.754 ± 0.008	0.316 ± 0.005	0.05 ± 0.02	0.5 ± 0.02
SIRMS	0.868 ± 0.004	34.4 ± 0.5	0.02 ± 0.008	26 ± 1	SIRMS	0.771 ± 0.008	0.302 ± 0.005	0 ± 0.004	0.52 ± 0.02
alvaDesc	0.848 ± 0.004	37.1 ± 0.5	0.05 ± 0.02	15 ± 1	alvaDesc	0.736 ± 0.008	0.328 ± 0.006	0 ± 0.01	0.48 ± 0.02
Transformer CNN	0.891 ± 0.003	31.2 ± 0.5	0.05 ± 0.01	25 ± 2	Transformer CNN	0.785 ± 0.008	0.292 ± 0.005	0.07 ± 0.03	0.57 ± 0.02
Consensus model					Consensus model				
	0.9 ± 0.003	30.1 ± 0.5	0.03 ± 0.01	21 ± 1		0.806 ± 0.007	0.279 ± 0.005	0 ± 0.005	0.54 ± 0.02

Table S4: Statistical coefficients calculated for the QSPR model for the PORPHYRINS set.

Absorption maxima position			Molar extinction coefficient	
Descriptors	Training, n=2241	Test, n=335	Descriptors	Training, n=946

	R^2	RMSE/nm	R^2	RMSE/nm		R^2	RMSE/log unit
Fragmentor	0.79 ± 0.01	5.5 ± 0.2	0.03 ± 0.02	3 ± 0.1	Fragmentor	0.49 ± 0.03	0.217 ± 0.007
MOLD2	0.73 ± 0.02	6.3 ± 0.2	0.02 ± 0.02	4.5 ± 0.1	Dragon7	0.49 ± 0.03	0.219 ± 0.006
PyDescriptor	0.73 ± 0.02	6.3 ± 0.2	0.05 ± 0.02	3.78 ± 0.1	RDKIT	0.49 ± 0.03	0.219 ± 0.006
QNPR	0.72 ± 0.02	6.3 ± 0.2	0.03 ± 0.01	2.7 ± 0.1	PyDescriptor	0.48 ± 0.03	0.219 ± 0.006
RDKIT	0.77 ± 0.01	5.8 ± 0.2	0.1 ± 0.02	5.6 ± 0.2	alvaDesc	0.49 ± 0.03	0.219 ± 0.006
SIRMS	0.77 ± 0.02	5.7 ± 0.2	0.09 ± 0.02	3.4 ± 0.1	MAP4	0.49 ± 0.03	0.221 ± 0.006
Transformer CNN	0.8 ± 0.01	5.4 ± 0.2	0.08 ± 0.02	5.2 ± 0.2	Transformer CNN	0.43 ± 0.03	0.231 ± 0.006
Consensus model					Consensus model		
	0.8 ± 0.01	5.4 ± 0.2	0 ± 0.005	2.26 ± 0.08		0.52 ± 0.02	0.209 ± 0.006

Table S5: Statistical coefficients calculated for the prediction of the test set compoundsa as function of the training set sizes for the PORPHYRIN sets.

Training set size, %	Absorption maxima position (n=2241)		Molar extinction coefficient (n=946)	
	R^2	RMSE	R^2	RMSE
10	0.51 ± 0.02	9.3 ± 0.3	0.31 ± 0.03	0.25 ± 0.006
20	0.59 ± 0.02	8.4 ± 0.3	0.41 ± 0.03	0.239 ± 0.007
30	0.61 ± 0.03	8.2 ± 0.3	0.41 ± 0.03	0.235 ± 0.007
40	0.69 ± 0.03	7.6 ± 0.4	0.42 ± 0.04	0.233 ± 0.008
50	0.74 ± 0.03	7 ± 0.4	0.42 ± 0.04	0.225 ± 0.008
60	0.74 ± 0.03	6.6 ± 0.4	0.47 ± 0.04	0.217 ± 0.009
70	0.77 ± 0.04	6.5 ± 0.5	0.5 ± 0.05	0.225 ± 0.01
80	0.71 ± 0.05	7.4 ± 0.6	0.36 ± 0.06	0.22 ± 0.01
90	0.71 ± 0.07	7 ± 1	0.55 ± 0.08	0.21 ± 0.02
5CV[b]	0.81 ± 0.01	5.2 ± 0.2	0.52 ± 0.03	0.213 ± 0.006

[a] The compounds that did not participate in the respective training sets were used as the test sets compounds.
[b] Results calculated using 5 fold cross-validation.

Table S6: Statistical coefficients calculated for the prediction of the test set compoundsa as function of the training set sizes for the compounds (n=335) measured in our laboratory.

Training set size, %	Absorption maxima position		Molar extinction coefficient	
	R2	RMSE	R2	RMSE
10	0.32 ± 0.06	1.45 ± 0.06	0.84 ± 0.02	0.167 ± 0.009
20	0.76 ± 0.03	1.18 ± 0.07	0.9 ± 0.01	0.128 ± 0.009
30	0.7 ± 0.04	0.97 ± 0.06	0.9 ± 0.02	0.116 ± 0.009

40	0.76 ± 0.05	0.89 ± 0.07	0.965 ± 0.006	0.089 ± 0.006
50	0.9 ± 0.02	0.6 ± 0.05	0.95 ± 0.01	0.09 ± 0.01
60	0.9 ± 0.02	0.61 ± 0.06	0.98 ± 0.004	0.048 ± 0.005
70	0.92 ± 0.02	0.65 ± 0.07	0.982 ± 0.005	0.057 ± 0.008
80	0.93 ± 0.02	0.46 ± 0.05	0.981 ± 0.009	0.06 ± 0.01
90	0.96 ± 0.02	0.42 ± 0.06	0.985 ± 0.007	0.045 ± 0.007
5CVb	0.93 ± 0.01	0.5 ± 0.03	0.989 ± 0.002	0.042 ± 0.004

[a] The compounds that did not participate in the respective training sets were used as the test sets compounds.
[b] Results calculated using 5 fold cross-validation.

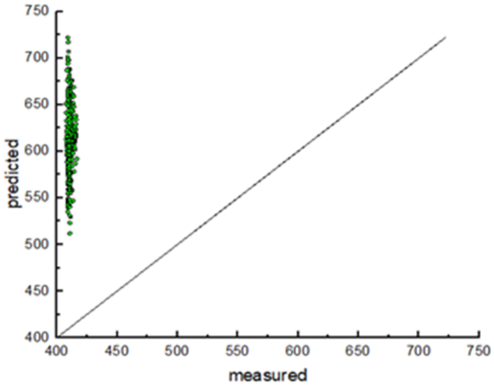

Figure S1: Distribution of experimental and predicted absorbance position values for the NOVEL set using the publicly available model (http://deep4chem.korea.ac.kr) developed by Joung et al27 (RMSE=200.08 and R2=0.01).

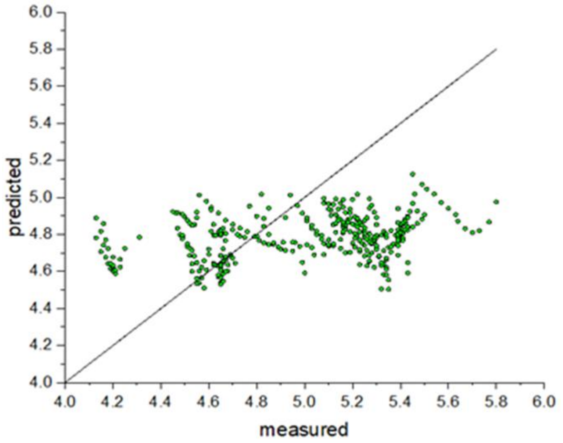

Figure S2: Distribution of the experimental and predicted values of the extinction coefficient for the NOVEL set according to the publicly available model (http://deep4chem.korea.ac.kr) developed by Joung et al27 (RMSE=0.89 and R2=0.10).

Experimental protocols

I.The condensation of 5,5′-unsubstituted dipyrromethane with aromatic aldehydes

The condensation reaction of 5,5′-unsubstituted dipyrromethane with aromatic aldehydes, with the intermediate formation of porphyrinogen and its subsequent oxidation with benzoquinone derivatives to porphyrin, was studied in

order to obtain 5,15-diarylsubstituted porphyrins with the necessary sensory properties. The product yield was optimized depending on the mutual position of substituents in the pyrrole fragment of dipyrromethane, the nature of the aromatic aldehyde, solvent, catalyst, and oxidant.

Synthesis of 5,15-diphenyl-2,3,7,8,12,13,17,18-octamethylporphyrin (1). In an atmosphere of carbon dioxide, a solution of 0.5 g (1.7 mmol) of 3,3,4,4'-tetramethyldipyrromethane and 0.2 ml (1.7 mmol) of benzaldehyde in 20 ml of ethanol was added over 10 min with stirring to a solution of 0.57 ml of trifluoroacetic acid in 40 ml of ethanol. The mixture was stirred for 3 h and neutralized with ammonia solution. Then 1 g (4.1 mmol) of tetrachlorobenzoquinone-1,2 in 15 ml of acetone was added and the mixture was stirred for 1.5 h. Ethanol was distilled off and the residue was washed with 180 ml of 10% sodium hydroxide solution. The precipitate was filtered off, dried, and chromatographed on silica gel with toluene. Yield: 112 mg (32.1%). R_f = 0.42 (silufol, toluene). ^{1}H NMR spectrum ($CDCl_3$, TMS): 10.12 s. (2H, meso-H), protons of the phenyl rings [7.98 m. (4H, o-H); 7.68 m. (6H, m-, p-H)], 2.41 s (24H, CH3), -2.42 br. with. (2H, NH). UV-vis spectrum, λ_{max} (dichloromethane): 628.0 (3.48), 576.0 (3.96), 539.0 (3.86), 507.0 (4.34), 410.2 (5.41).

5,15-Diphenylporphyrins (2-113) were synthesized similarly to (1) by the condensation reaction of 5,5-unsubstituted dipyrromethanes with aromatic aldehydes (Scheme 1), with the intermediate formation of porphyrinogen and its subsequent oxidation with organic oxidants to porphyrin using catalysts and a solvent of various nature.

Scheme 1

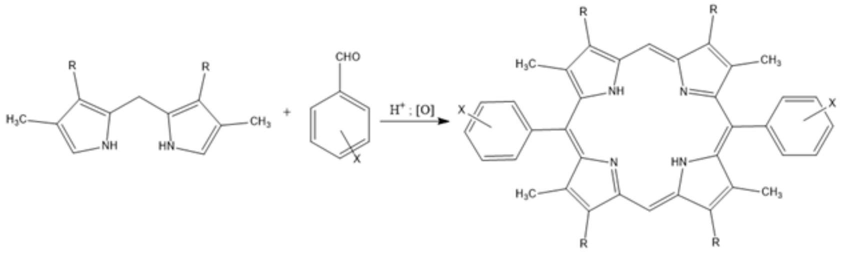

1 - 112

See also supplementary materials Excel file with chemical structures.

II. The reaction of template cyclotetramerization of 5,5'-dicarboxydipyrromethanes with aromatic aldehydes.

The reaction of template cyclotetramerization of 5,5'-dicarboxydipyrromethanes with aromatic aldehydes in pyridine in the presence of zinc cation was studied. The product yield was optimized depending on the length of alkyl substituents on the pyrrole fragment of dipyrromethane and the nature of the aromatic aldehyde.

Synthesis of 5,15-diphenyl-2,3,7,8,12,13,17,18-octamethylporphyrin (1). A mixture of 0.5 g (1.3 mmol) of 5,5'-dicarboxy-3,3', 4,4'-tetramethyldipyrromethane, 0.63 g (3.4 mmol) of anhydrous zinc acetate, 0.49 g (4.9 mmol) of benzaldehyde, 8 ml of pyridine and 0.48 ml (4.8 mol) nitrobenzene was kept in a sealed ampoule at 180°C for 14 h. The reaction mixture was cooled, refluxed with water for 30 min, and filtered. The precipitate was dried, dissolved in a small amount of chloroform. Trifluoroacetic acid was added to the solution and stirred at room temperature for 30 min. The acid was neutralized with an ammonia solution, chloroform was evaporated to a minimum volume and chromatographed on silica with benzene. Yield: 100 mg (39.8%). R_f = 0.42 (silufol, toluene). ^{1}H NMR spectrum ($CDCl_3$, TMS): 10.12 s. (2H, meso-H), protons of the phenyl rings [7.98 m (4H, o-H); 7.68 m (6H, m-, p-H)], 2.41 s (24H, CH3), -2.42 br.s (2H, NH). UV-vis spectrum, λ_{max} (dichloromethane): 628.0 (3.48), 576.0 (3.96), 539.0 (3.86), 507.0 (4.34), 410.2 (5.41).

5,15-Diphenylporphyrins (113-242) were synthesized (Scheme) similarly to (1) by the reaction of template cyclotetramerization of corresponding 5,5'-dicarboxy-dipyrromethanes with aromatic aldehydes in pyridine in the presence of a zinc cation in a sealed ampoule at 180°C for 14 h.

Scheme 2

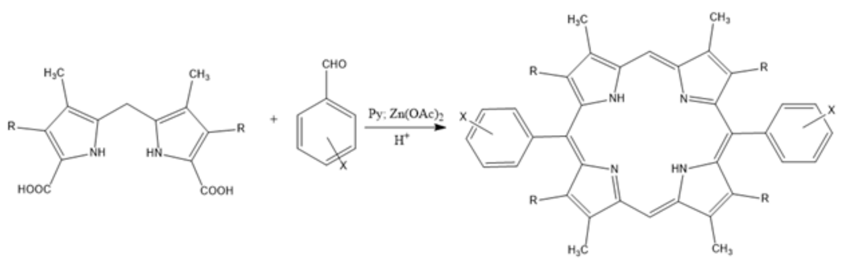

113 - 232

See also supplementary materials Excel file with chemical structures.

III. Synthesis of Zn-5,15-diphenyl-2,3,7,8,12,13,17,18-octamethylporphyrin (243). A solution of 100 mg of 5,15-diphenyl-2,3,7,8,12,13,17,18-octaethylporphyrin ligand and 200 mg of zinc acetate in 100 ml of dimethylformamide was refluxed for 30 minutes, cooled, 100 ml of chloroform was added and poured into 300 ml of water. The organic layer was separated, washed with water, the solution was evaporated to a minimum volume and chromatographed on aluminum oxide, eluting the Zn-porphyrin complex with a mixture of chloroform-hexane, 40:1. Yield: 94.5%. R_f=0.42 (silufol, hexane-acetone, 16:1). ^{1}H NMR spectrum ($CDCl_3$, TMS): 10.09 s. (2H, meso-H), protons of the phenyl rings [7.94 m (4H, o-H); 7.63 m (6H, m-, p-H)], 2.36 s (24H, CH3). UV-vis spectrum, λ_{max} (dichloromethane): 570.0 (3.66), 539.0 (3.86), 409.0 (4.61).

Zn-complexes 244-335 were synthesized similarly (243) by the reaction of complexation of the corresponding diphenylporphyrins with zinc acetate in DMF.

Scheme 3

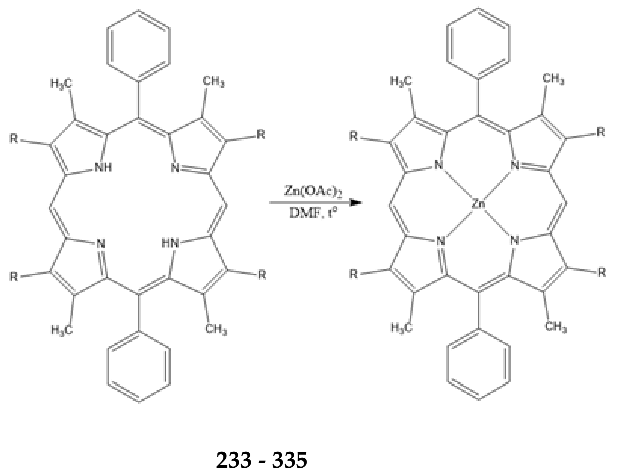

233 - 335

See also supplementary materials Excel file with chemical structures.

UV-vis spectra of porphyrins **1-335** in dichloromethane (λ_{max}, nm / logε) are provided in the supplementary materials Excel file with chemical structures.
